# lncRNA XIST promotes the progression of laryngeal squamous cell carcinoma by sponging miR-144 to regulate IRS1 expression

**DOI:** 10.3892/or.2021.8208

**Published:** 2021-10-21

**Authors:** Chang-Lei Cui, Yi-Ning Li, Xiang-Yan Cui, Xin Wu

Oncol Rep 43: 525-535, 2020; DOI: 10.3892/or.2019.7438

The authors wish to retract their research article entitled ‘lncRNA XIST promotes the progression of laryngeal squamous cell carcinoma by sponging miR-144 to regulate IRS1 expression’, published in *Oncology Reports* 43: 525–535, 2020. They have found that, having repeated several of the experiments, XIST expression does not appear to affect LSCC cell apoptosis. In addition, some of their original data had been lost due to computer damage. Therefore, all authors are in agreement that this paper published in *Oncology Reports* should be retracted to maintain the integrity of the scientific record.

All the named authors on the paper agree to this retraction, and they sincerely apologize for any inconvenience that might result from the retraction of this article.

